# High-Performance CP Magneto-Electric Dipole Antenna Fed by Printed Ridge Gap Waveguide at Millimeter-Wave

**DOI:** 10.3390/s24248183

**Published:** 2024-12-21

**Authors:** Zahra Mousavirazi, Mohamed Mamdouh M. Ali, Peyman PourMohammadi, Peng Fei, Tayeb A. Denidni

**Affiliations:** 1 Centre-Energie Materiaux et Telecommunications, Institut National de la Recherche Scientifique, Montreal, QC H5A 1K6, Canada; zahra.mousavirazi@inrs.ca (Z.M.); tayeb.denidni@inrs.ca (T.A.D.); 2 Electrical Engineering Department, Assiut University, Assiut 71515, Egypt; mohamed.ali@ieee.org; 3 Beijing Institute of Radio Metrology and Measurement, Beijing 100854, China; feipeng@ieee.org

**Keywords:** circular polarization (CP), magneto-electric (ME) dipole, 5G and 6G applications, wideband, printed ridge gap waveguide (PRGW), millimeter-wave (mm-wave)

## Abstract

This paper presents a high-performance circularly polarized (CP) magneto-electric (ME) dipole antenna optimized for wideband millimeter-wave (mm-wave) frequencies, specifically targeting advancements in 5G and 6G technologies. The CP antenna is excited through a transverse slot in a printed ridge gap waveguide (PRGW), which operates in a quasi-transverse electromagnetic (Q-TEM) mode. Fabricated on Rogers RT 3003 substrate, selected for its low-loss and cost-effective properties at high frequencies, the design significantly enhances both impedance and axial ratio (AR) bandwidths. The antenna achieves an impressive impedance bandwidth of 31% (25.24–34.50 GHz) and an AR bandwidth of 24.9% (26.40–33.91 GHz), with a peak gain of up to 8.4 dBic, demonstrating a high cross-polarization level. The experimental results validate the high-performance characteristics of the antenna, making it a robust candidate for next-generation wireless communication systems requiring CP capabilities.

## 1. Introduction

Millimeter-wave (mm-wave) technology is rapidly emerging as a pivotal element in advancing the next-generation of wireless communication systems, such as 5G and the forthcoming 6G networks [1,2,3,4,5]. Operating at these higher frequencies offers the dual benefits of expansive bandwidths and the potential for ultra-fast data rates, making mm-wave a highly attractive option for future wireless applications. Engineers and researchers are pushing the boundaries of what is possible in wireless network performance, capacity, and efficiency to fully leverage the capabilities of mm-wave technology [6].

The transition to mm-wave frequencies is primarily driven by the need for higher data rates and lower latency, alongside the availability of previously under-utilized mm-wave spectrum. However, the deployment of mm-wave technologies introduces several substantial challenges. These include overcoming significant path losses, signal attenuation from atmospheric conditions, and physical blockages such as buildings and foliage [7,8,9,10].

The design and development of mm-wave antenna systems must address factors such as impedance bandwidth, efficiency, size, material selection, and manufacturing precision to ensure effective communication links [11,12,13,14,15,16]. High-gain antennas are particularly crucial in mm-wave applications due to their ability to focus transmitted signals into a narrow beam thus concentrating energy in the intended direction. This focused beamforming helps to compensate for the high propagation losses and attenuation typical of mm-wave signals. By enhancing the range and reliability of communication links, high-gain antennas also optimize power usage, reduce interference, and mitigate environmental disturbances, which altogether boost the overall system performance.

Moreover, circularly polarized (CP) antennas are proving to be indispensable in the realm of mm-wave communications. Their ability to mitigate multipath interference and improve signal reception in dynamic environments enhances their utility in complex wireless landscapes. CP antennas are favored for their capability to minimize the adverse effects of polarization mismatch and reflections, which are common challenges in high-frequency communication scenarios. With the ongoing development of CP antennas featuring wideband capabilities and high-gain, these antennas are set to play a crucial role in the effective deployment of mm-wave technology, particularly in mobile and fixed wireless applications [17,18,19,20,21,22,23].

Techniques for designing CP antennas are classified into single-fed and dual/multi-fed types. The single-fed CP antennas are less complicated due to the simple feeding structure but are limited by the relatively narrow axial ratio (AR) bandwidth. Even though multi-fed CP antennas improve the operating bandwidth effectively, they increase the structure complexity owing to the presence of dual or multi ports. Hence, they are not suitable for next-generation space-limited applications. The CP antennas with a single-feed are mostly preferred to reduce the loss and achieve a compact size. Various technologies to improve the operating bandwidth of single-fed CP antennas such as patch antenna, L- and U-shaped slot antenna [24,25], cavity-backed slot antenna [26], helix and spiral antenna [17,27,28], magneto-electric (ME) dipole antenna [29,30], and S-, L- and cross-shaped dipole antenna [31,32,33] were explored for mm-wave applications [34]. Among these, the ME dipole antenna has emerged as the preferred choice for CP antenna design due to its exceptional efficiency in circular polarization and its ability to maintain stable signal transmission over a wide range of frequencies [35,36].

The ME-dipole element, serving as a complementary source antenna, is renowned for its expansive operational bandwidth and excellent radiation properties, making it ideal for applications that demand robust and efficient wireless communications [37]. By integrating the properties of both magnetic and electric dipoles, the ME-dipole antenna offers outstanding circular polarization and delivers stable, strong signal transmission across a wide frequency range, which is essential for modern high-frequency communication systems. Research on designing ME dipole antennas for next-generation mm-wave applications highlights several innovative feeding techniques to enhance performance, especially in circular polarization [2,14].

Key approaches include L-shaped probe feeds, which stabilize performance across wide frequency ranges, and microstrip line feeds that simplify the design while effectively supporting circular polarization. Additionally, some designs incorporate metallic walls around the ME-dipole elements, paired with a microstrip power divider for feeding, significantly expanding the axial ratio bandwidth and improving polarization. These methods, including combinations of substrate-integrated waveguide (SIW) and microstrip techniques, underscore the adaptability of ME-dipole antennas in meeting the rigorous demands of modern high-frequency communication systems [36,38,39]. In [29,30], low-profile ME-dipole antennas exited by a SIW feed network were reported to achieve a wide impedance bandwidth. However, both antennas had narrow axial ratio (AR) bandwidths of approximately 16% and 13%, respectively. To improve the AR bandwidth, a CP ME-dipole antenna was proposed in [40], which involved designing a double-sided printed electric dipole based on SIW. This led to an increased AR bandwidth of about 36.6% but at the expense of increased antenna size. Moreover, with the increase in frequency, the transmission loss of the SIW feeding networks is inevitable.

A novel waveguide structure known as the ridge gap waveguide (RGW) was developed to address these challenges, targeting high-frequency applications in 5G communication systems [41]. This innovative approach overcomes the limitations of traditional waveguides by facilitating lower-loss transmission and broader bandwidth capabilities, making it an ideal choice for modern high-frequency communication systems.

Over the past decade, significant research has focused on RGW technology to develop high-efficiency. These low-loss transmission lines minimize complexity by eliminating the need for metal connections, particularly at high frequencies. Introduced in 2009 as a novel low-loss transverse electromagnetic (TEM) guiding structure, RGW was further developed into its printed version, the printed ridge gap waveguide (PRGW), by 2011 [42,43]. This iteration offers an economical solution for mm-wave frequencies and employs a quasi-transverse electromagnetic mode of propagation, enhancing its suitability for applications requiring high precision and efficiency in compact forms.

PRGW technology is distinguished by its low-loss and minimal dispersion compared to traditional printed circuit board (PCB)-based structures, making it highly effective for high-frequency applications. It enables the integration of planar transmission lines with other circuit components, which is crucial for designing low-cost and low-profile physical structures. In RGW setups, electric and magnetic waves are tightly confined between two metal walls with open sidewalls, allowing propagation through the air gap. This unique configuration significantly reduces losses compared to SIW technologies, where waves traverse through both air and the host dielectric material, incurring higher losses [44,45,46].

In [47], a high-gain CP ME-dipole antenna element fed by PRGW was implemented to excite a high-mode cavity. However, only narrow impedance and AR bandwidths of about 13% were obtained in [12]; a high-efficiency CP ME-dipole based on PRGW technology using a three-layer dual-polarized split-ring resonator lens has achieved a sufficient gain above 10dBic. However, the proposed structure has a narrow impedance and AR bandwidth. Additionally, using a three-layer lens above the antenna adds more losses and difficulties in fabrication, especially for large antenna arrays. Although a CP antenna element designed in [18] has a low-profile structure, it suffers from a narrow AR bandwidth of 10%, which is insufficient for 5G applications.

This paper proposes a wideband and compact CP antenna element fed by PRGW technology designed with a single-feed configuration at 28 GHz. Circular polarization in this antenna is achieved through the upper part of the antenna as a magneto-electric dipole antenna. In similar works, circular polarization is created through dual-feed configuration, which adds design complexity, or via an additional layer placed above the magneto-electric dipole antenna, introducing extra complexity and height to the antenna. We have employed a fork-shaped feed structure to increase the bandwidth, which has led to promising results alongside the PRGW technology. The simulated and experimental results agree well and indicate a wide impedance bandwidth of about 31% from 25.24 to 34.50 GHz. It is worth noting that the main objective of this paper is to improve the bandwidth of the CP antenna by integrating a novel ME-dipole antenna into a single-layered substrate. This allows for creating a wideband CP antenna element without needing a complex feed network or additional layers. It is also worth mentioning that all antenna layers are designed using Rogers RT 3003, a high-performance substrate optimized for high-frequency applications. With a dielectric constant (ε_r_) of 3, this material is ideal for scenarios that demand minimal signal delay and phase shift, such as 5G network antennas. Rogers RT 3003 is renowned for its low dielectric loss, which enhances efficiency by minimizing power loss at higher frequencies. Additionally, its combination of affordability and high-performance makes it a compelling choice for designing cost-effective, yet highly efficient, high-frequency components.

## 2. ME-Dipole Antenna Design Procedure

**Figure 1:**
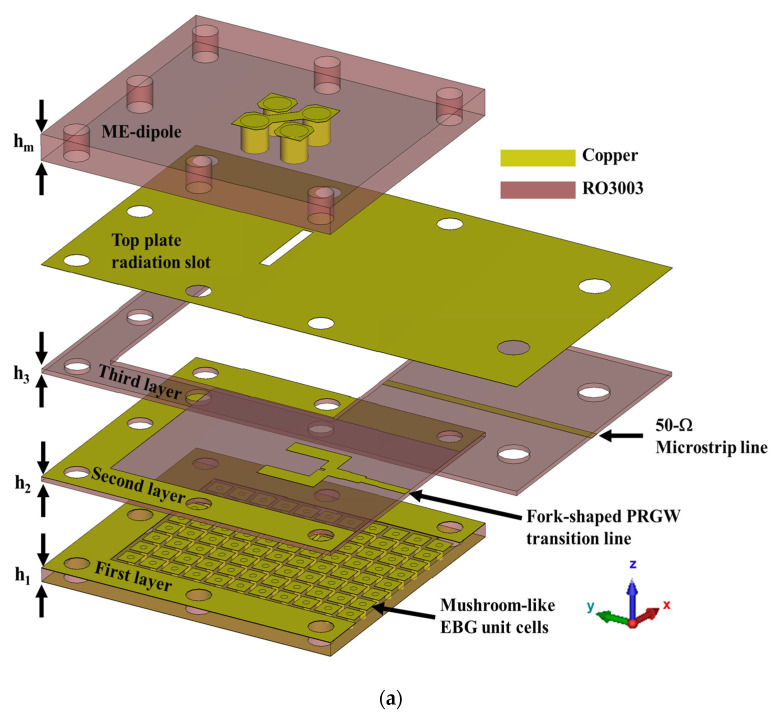

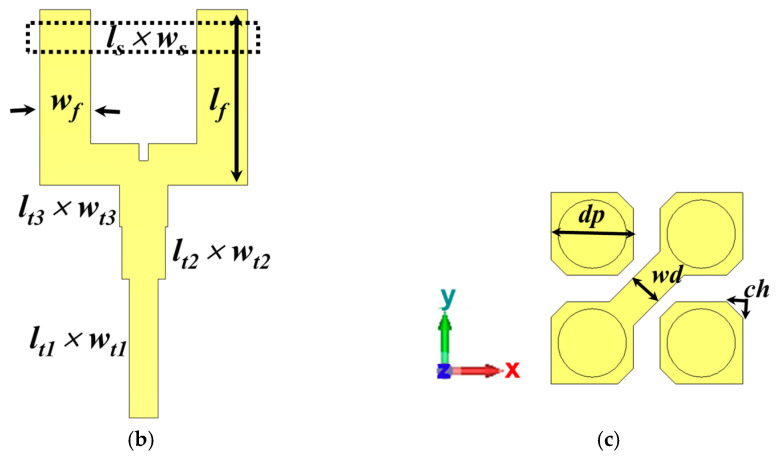
(**a**) The perspective view of the exploded CP antenna, (**b**) the top view of the fork-shaped feed network, and (**c**) the top view of the ME-dipole.

The 3-D view of the proposed mm-wave CP ME-dipole antenna is shown in Figure 1a. From the illustration, the CP antenna comprises a low-loss PRGW feed network and a high-performance CP ME dipole antenna. The fork-shaped ridge-line is printed on the second layer of an RT3003 substrate, which has a relative dielectric constant (ε_r_) of 3 and a thickness (h_2_) of 0.254 mm. In this antenna structure, a fork-shaped feedline is utilized, offering distinct advantages for enhancing performance. This design significantly improves impedance matching over a broad frequency range, facilitating efficient power transfer and broader bandwidth. Additionally, the fork-shaped feed enhances the uniformity of current distribution across the antenna, leading to improved radiation patterns and antenna gain [2]. The dimensions of the fork-shaped ridge-line are demonstrated in Figure 1b.

The fork-shaped ridge-line is connected to the antenna input via a 50 Ω microstrip line (MS). A 50 Ω microstrip line, with a width of 0.63 mm, is positioned on the bottom of the third layer, which has the same thickness as the second layer. This line is used to transfer power from the antenna input towards the ridge-line. Additionally, the third layer serves as a spacer, maintaining a constant air gap (h_gap_ = 0.254 mm), less than a quarter wavelength (λ/4), between the ridge-line and the upper ground plane. This arrangement assists with the propagation of the quasi-TEM mode. A transverse narrow radiating slot, measuring 5.2 mm by 0.7 mm, is etched on the metallic ground plane located on the top plate. This slot is designed to excite the ME-dipole antenna structure. The geometrical specifications of the ME-dipole antenna structure are depicted in Figure 1c.

### 2.1. Printed Ridge Gap Waveguide Design

**Figure 2:**
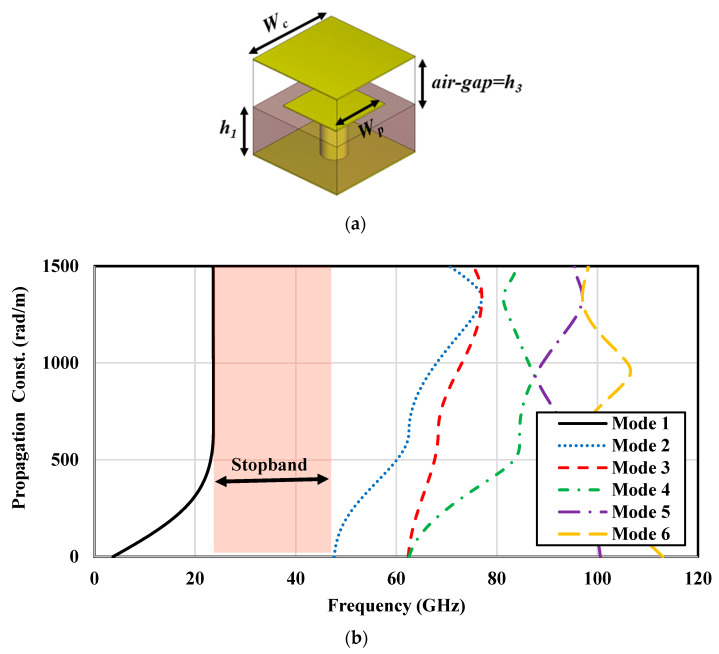
(**a**) Configuration and (**b**) dispersion diagram of proposed square PRGW unit cell.

The low-loss PRGW is formed by periodic mushroom-like unit cells printed on the RT3003 substrate acting as electromagnetic bandgap (EBG) cells that are connected to the ground plane through metalized vias [48,49]. The periodic arrangement of EBG unit cells functions as an Artificial Magnetic Conductor (AMC) surface, which emulates Perfect Magnetic Conductor (PMC) behavior across a specific frequency bandwidth. By maintaining an air gap height smaller than λ/4, electromagnetic waves (EMs) can propagate between the upper ground plane and the ridge. This configuration effectively suppresses wave propagation outside the ridge between the AMC and the ground plane, creating a distinct stopband. The dimensions of an EBG unit cell and its dispersion, which is obtained using the computer simulation technology (CST) (Eigen-mode solver), are depicted in Figure 2. This analysis reveals a propagation constant of zero at frequencies between 24 GHz and 48 GHz, highlighting a critical frequency range for operational effectiveness. The unit cell is designed with a square shape to simplify the engineering process, enhancing the ease of integration and design consistency in practical applications [50].

### 2.2. Operating Principle

**Figure 3:**
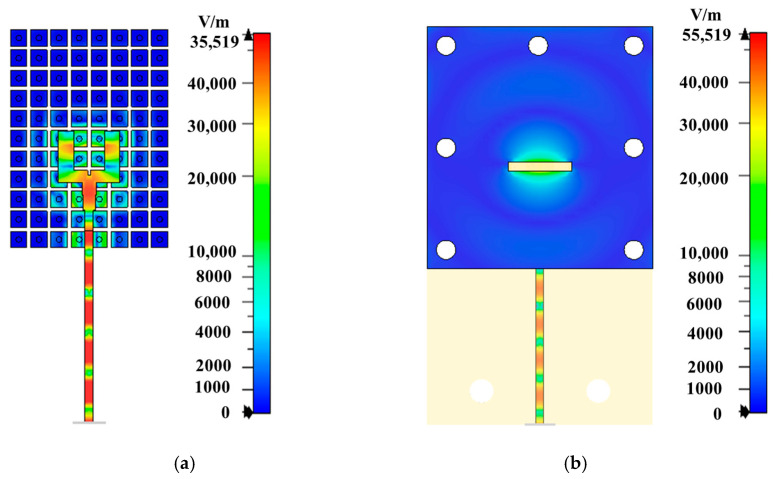
The simulated E-field distribution (**a**) of the fork-shaped feed network and (**b**) around the radiating slot.

Figure 3 shows the E-field distribution in the fork-shaped feed network and around the radiating slot, clearly demonstrating that the signal is transferred from the transition line to two equal signals of the fork-shaped ridge-line without any leakage from the feeding line and the field is confined along the ridge-line feeding the slot antenna with maximum radiation. The final layer of the Rogers RT 3003 substrate, which has a thickness of hm = 1.524 mm and serves as the radiation layer, consists of four horizontally oriented defected square patches. These patches are connected to the ground plane through vertical metallic vias. Each pair of grounded horizontal patches functions as a planar electric dipole (E-dipole), while the aperture between the patches serves as a magnetic dipole (M-dipole). The ME-dipole antenna operates by simultaneously exciting these magnetic and electric dipoles through the slot thereby generating the ME-dipole mode with linear polarization (LP) [51,52].

**Figure 4:**
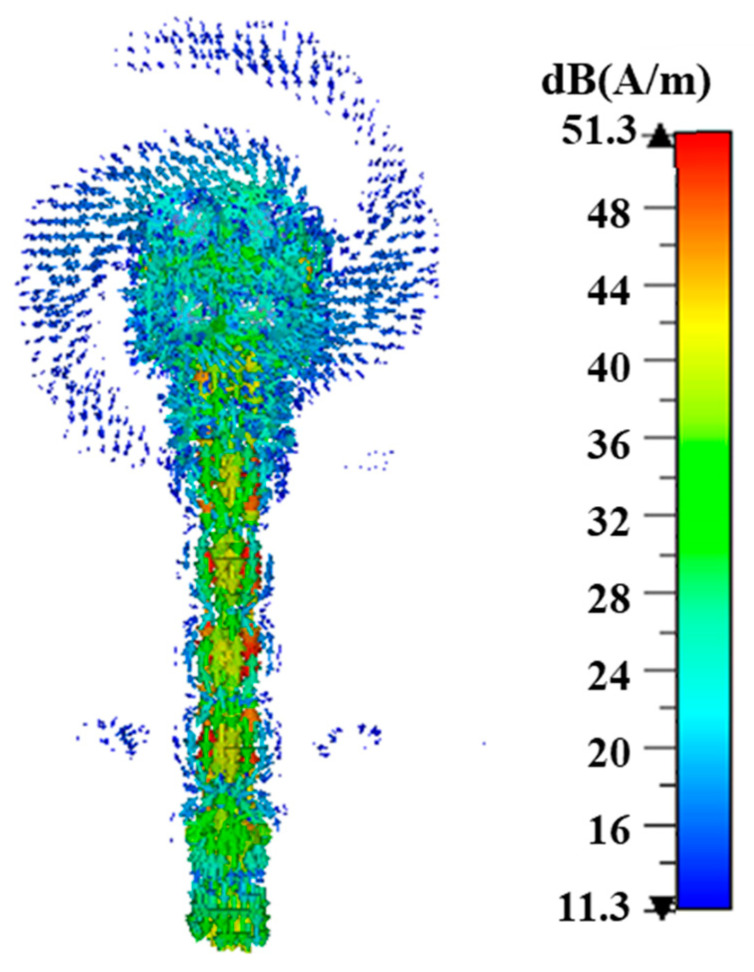
The rotation surface currents in the antenna.

To achieve the desired CP performance, a narrow strip with a width of 0.8 mm connects the two inner corners of the diagonal patches, forming a bridge over the slot. Additionally, the corners of the other two defected patches are cut, as depicted in Figure 1c. This configuration allows for the energy from the exciting slot to be coupled and excites another mode of ME-dipole in the orthogonal direction. By optimizing the dimensions of the horizontal defected patches and apertures, a 90° phase difference between the two modes is achieved, which is necessary to generate CP radiation. Figure 4 clearly shows how the current, after radiation from the dipole antenna, is rotated, confirming the circular polarization performance of the designed antenna at 28 GHz.

**Figure 5:**
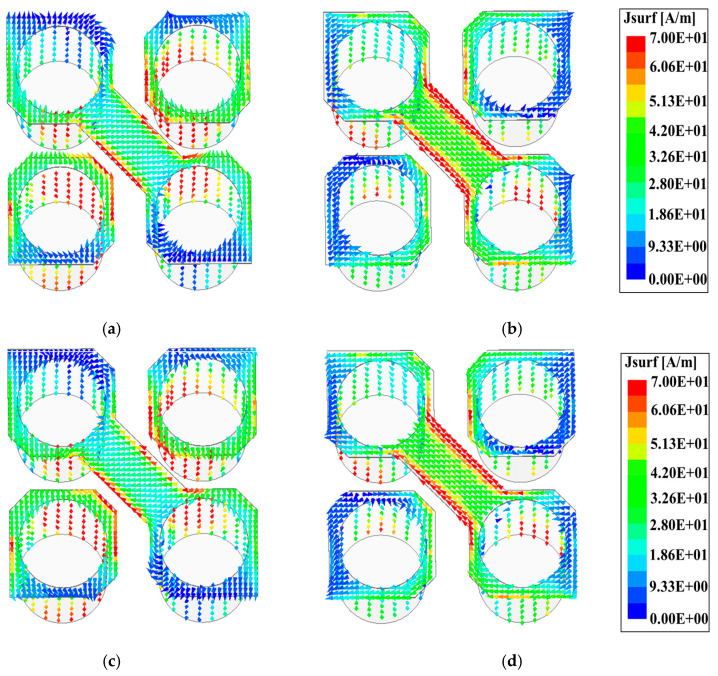
The simulated current distributions over the ME-dipole antenna at a period of T for (**a**) t = 0, (**b**) t = T/4, (**c**) t = T/2, and (**d**) t = 3T/4.

To clarify the operating principle of the designed CP antenna, the simulated surface current distributions over the ME-dipole antenna are illustrated in Figure 5. These distributions are shown at the operating frequency of 28 GHz over a period of (T).

From Figure 5, it can be observed that at time t = 0 and t = T/2, the surface current distributions are directed along the y-direction and x-direction on the horizontal patches and the vertical vias, respectively. That indicates that the electric dipole in the y-direction and the magnetic dipole in the x-direction are excited at the same time. In addition, at t = T/4 and t = 3T/4 the direction of the surface current distributions changes by 90^o^. This means that the magnetic dipole is excited in the y-direction while the electric dipole is excited in the x-direction. Moreover, it is evident that as the time t increases from 0 to 3T/4, the surface current rotates clockwise, resulting in LHCP radiation.

### 2.3. Parametric Study

To better understand how various geometrical parameters of the ME-dipole antenna enhance the performance of the CP antenna, a series of parametric studies were conducted. These studies focused on the impact of critical parameters, such as strip thickness (w_d_), cut depth of horizontal patches (ch), and width of the horizontal patches (d_p_), on the reflection coefficient (|S_11_|) and AR. Each parameter was varied while keeping the others at their optimized values, as listed in Table 1.

**Figure 6:**
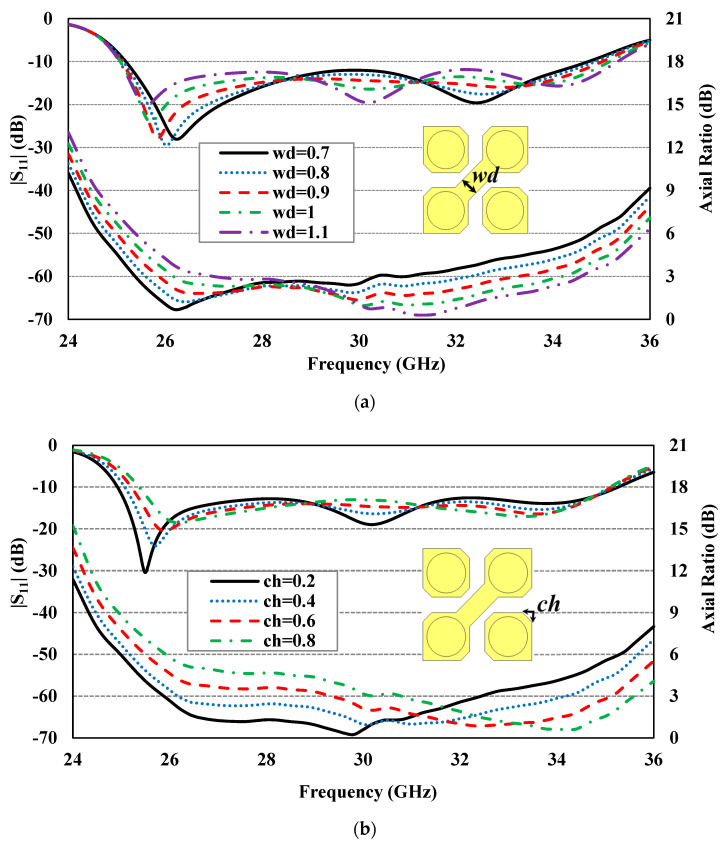

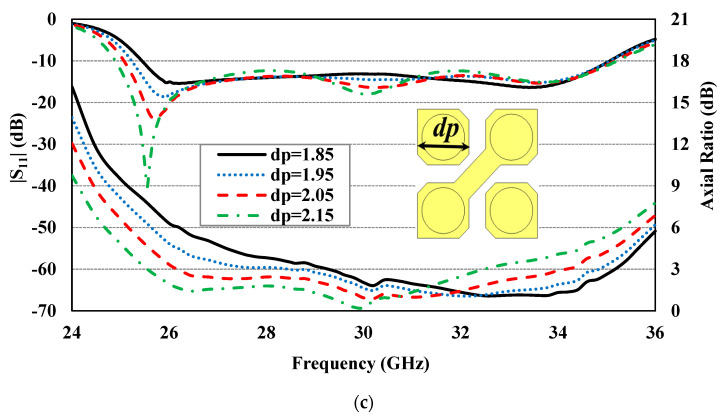
The simulated |S_11_| and AR of the designed CP antenna for (**a**) w_d_, (**b**) ch, and (**c**) d_p_ (unit: mm).

Figure 6a presents the simulated results for |S_11_| and AR at different widths of the middle diagonal strip connecting the two defected patches (w_d_), ranging from 0.7 to 1.1 mm. It is observed that increasing wd shifts the impedance and AR bandwidth to higher frequencies.

Figure 6b illustrates the effect of increasing the cut depth of horizontal patches (ch) from 0.2 to 0.8 mm, which significantly shifts the AR bandwidth to higher frequencies while maintaining a relatively stable |S_11_|.

Finally, Figure 6c displays the simulation results for |S_11_| and AR at different widths of the horizontal patches (d_p_). Adjusting dp from 1.85 to 2.15 mm improves the AR performance and shifts it to lower frequencies, resulting in a better |S_11_| match.

## 3. Experimental Validation

**Figure 7:**
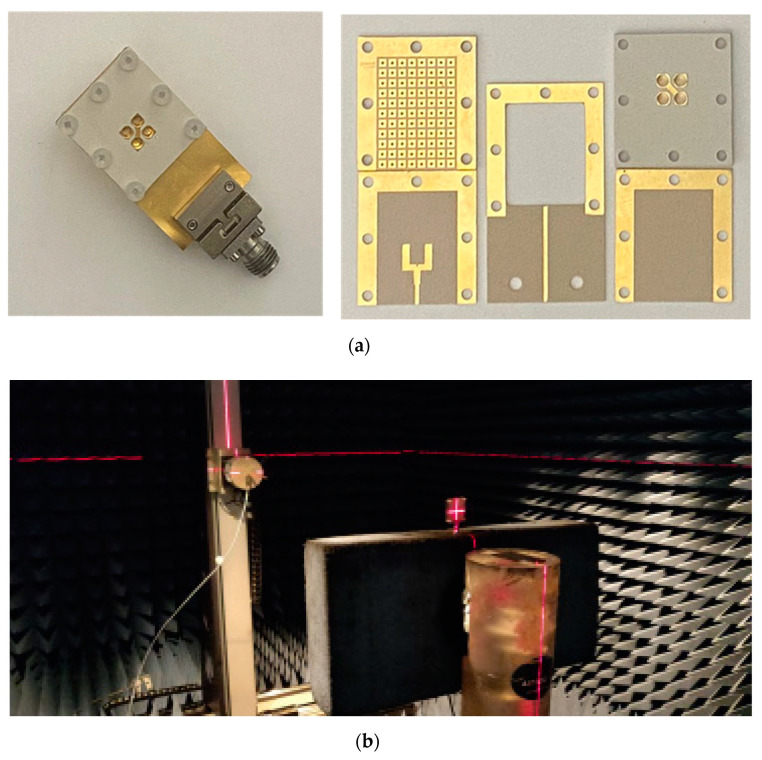
Photographs of fabricated CP PRGW ME-dipole antenna: (**a**) fabricated prototype and (**b**) far-field measurement setup.

A prototype was fabricated to experimentally validate the performance of the designed CP antenna. Figure 7 shows the photographs of the fabricated prototype, which was assembled by fabricating each layer separately and then joining them using multiple plastic screws (M2). The 50 Ω microstrip line on the third layer was extended to connect a 2.92 mm (K) end-launch connector.

To ensure the accuracy of the experimental results, two prototypes were fabricated. The first prototype, shown in Figure 7, was assembled using screws to connect the layers, ensuring a tightly secured structure. In the second prototype, the layers were glued together under pressure in a machine setup. However, this process may have caused slight bending of the layers due to the applied pressure, which could have slightly affected the characteristics of the antenna. While the measurement results from both prototypes were close, the first prototype showed slightly better performance and matched the simulation results more closely. This is likely due to the controlled assembly method in the first prototype, which avoided the potential deformation caused by the gluing process. As a result, we relied on the measurements from the first prototype for the final results presented in this paper.

Additionally, the radiation measurements were conducted in an antenna chamber designed for accurate far-field testing. The chamber provided a controlled environment, free from external interference, ensuring reliable results that accurately reflect the performance of the antenna under ideal conditions, as shown in Figure 7b.

**Figure 8:**
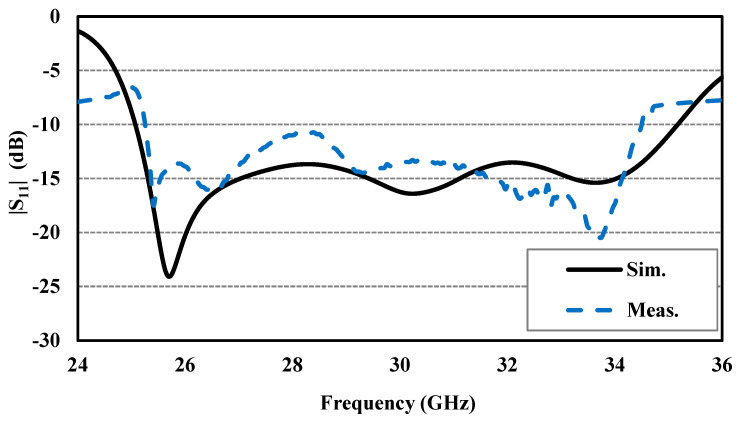
The simulated and measured reflection coefficient (|S_11_|) of the designed CP antenna.

**Figure 9:**
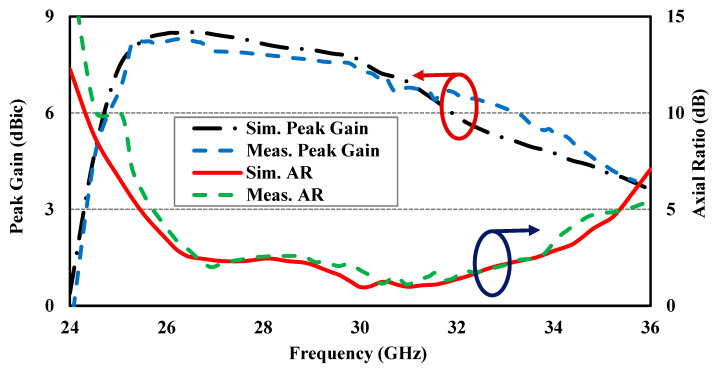
The simulated and measured peak gain and axial ratio of the designed CP antenna.

**Figure 10:**
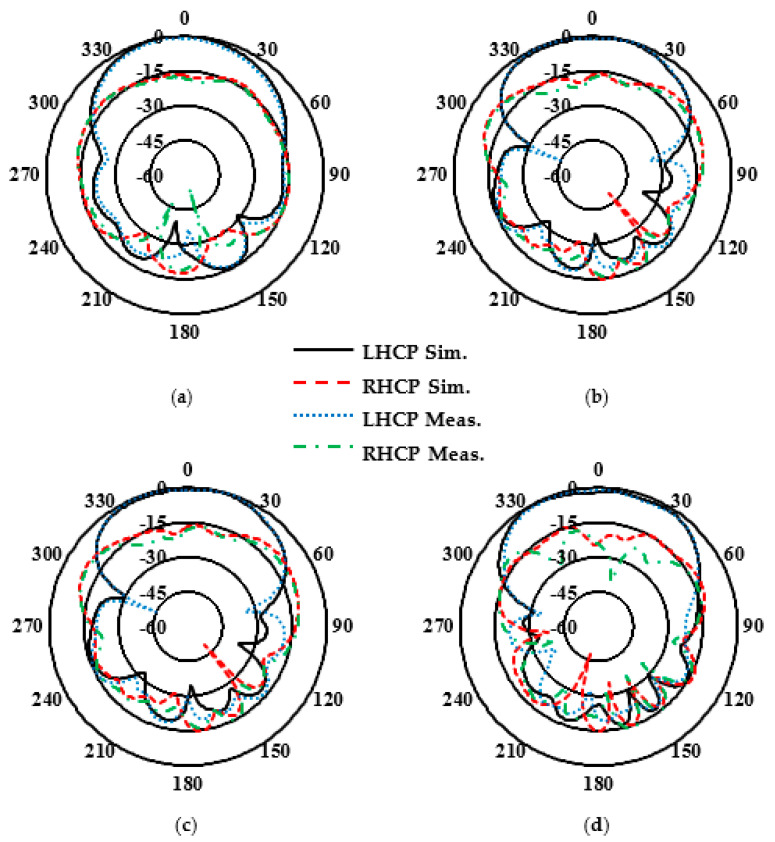
The simulated and measured normalized LH/RHCP radiation pattern of the CP PRGW ME-dipole antenna in the xoz plane at (**a**) 28 GHz and (**b**) 32 GHz, and in the yoz plane at (**c**) 28 GHz and (**d**) 32 GHz.

**Figure 11:**
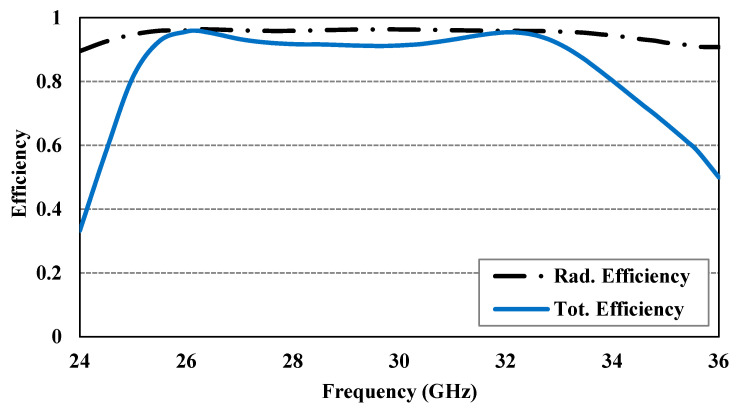
The simulated efficiency of the CP PRGW ME-dipole antenna.

The simulated and experimental reflection coefficients shown in Figure 8 are in good agreement, although there may be minor differences due to assembly and fabrication errors. The Agilent 8722ES Vector Network Analyzer was used to measure the reflection coefficient characteristics, confirming that the designed CP antenna is well matched (for |S_11_| below −10 dB) for a wideband impedance bandwidth of approximately 31.0%, ranging from 25.24 to 34.5 GHz. Figure 9 presents the simulated and experimental peak gain and AR of the proposed CP antenna, demonstrating the measured peak gains of about 7.8 and 6.65 dBic at 28 and 32 GHz, respectively. Further, the designed CP antenna has a 3 dB AR bandwidth wider than 24.9%, ranging from 26.40 to 33.91 GHz. The normalized simulated and experimental co-polarization (LHCP) and cross-polarization (RHCP) radiation patterns of the antenna are plotted in Figure 10 at two operating frequencies, 28 and 32 GHz, in both xoz- and yoz-planes. It can be observed that the designed CP antenna radiates as an LHCP antenna with a stable directional radiation pattern in the +z direction. On both xoz- and yoz-planes for both frequencies (28 and 32 GHz), the isolation between co- and cross-polarizations is over 15 dB in the broadside direction. The radiation and total efficiencies of the CP antenna are depicted in Figure 11. The antennas, which are fed by PRGW technology, exhibit efficiencies better than 96%. This high-efficiency is attributed to the minimal transmission losses characteristic of PRGW technology at high frequencies.

## 4. Discussion

To highlight the superiority of the presented antenna performance, a comparison with other reported CP antennas designed by different feed network technologies is summarized in Table 2. Low-cost PCB technology was used in all listed planar antennas for the advantage of easy integration and fabrication at high frequencies. In [12], a ME-dipole antenna using a meta-surface was utilized to improve the gain and the radiation efficiency of the antenna but suffered from narrow impedance and AR bandwidths. The CP antenna in [17] produced a wide impedance bandwidth, but the gain of the antenna was low and had the problem of a bulky profile due to the usage of the spiral antenna. The impedance and AR bandwidths of the CP antenna based on PRGW technology reported in [18] were not wide enough. Compared to the SIW-fed CP ME-dipole antennas in [29,30,51], the proposed antennas fed by PRGW obtain greater efficiencies, more than 96%, because of minimal transmission losses at high frequencies. In summary, the results emphasize that the designed low-profile and high-performance CP antenna in terms of wider impedance and 3 dB AR bandwidths as well as acceptable gain is a potential candidate for the next-generation mm-wave communications with CP capabilities.

## 5. Conclusions

A planar CP ME-dipole antenna was proposed in this section for mm-wave wideband applications operating at Ka-band. The ME-dipole antenna was excited by a transverse narrow slot that was coupled to a fork-shaped low-loss PRGW feed network. To achieve CP performance, an additional metallic strip was introduced to connect the inner corners of the defective patches by creating two orthogonal modes with similar amplitudes and a 90◦ phase difference. The fabricated antenna demonstrates excellent CP performance, including wide impedance and AR bandwidths of 31% and 24.9%, respectively. An experimental peak gain of 7.8 dBic at 28 GHz was also achieved. The results demonstrate superiority over similar works. With these features, the proposed antenna is an attractive candidate for future 5G applications due to its wideband and low-loss capabilities.

## Figures and Tables

**Table 1:** Optimized dimensions of proposed CP ME-dipole antenna (units: mm).

Parameters	h_1_	h_2_	h_3_	h_m_	l_s_	w_s_	w_f_
Values (mm)	0.762	0.254	0.254	1.524	5.2	0.7	1.13
Parameters	l_t1_	w_t1_	l_t2_	w_t2_	l_t3_	w_t3_	t_s_
Values (mm)	3.09	0.63	1.16	0.96	0.92	1.1	0.2
Parameters	d_p_	ch	l_f_	w_d_	_Wp_	_Wc_	Air gap
Values (mm)	2.05	0.4	3.89	0.8	1.23	1.5	0.254

**Table 2:** Performance comparison between CP antenna configurations.

Ref.	Frequency (GHz)	Antenna Type	Feen Network Technology	Impedance BW (−10 dB)	3dB ARBW	Gain (dBic)
[12]	28	ME-dipole	PRGW	24.24%	7.4%	10.7
[17]	34	Spiral	PRGW	37.2%	23.2%	7.2
[18]	28	Aperture	PRGW	15.6%	10%	9
[29]	28	ME-dipole	SIW	24.2%	16.5%	-
[30]	25	ME-dipole	SIW	32.6%	12.8%	7.8
[51]	60	ME-dipole	SIW	28.8%	25.9%	9
This work	28	ME-dipole	PRGW	31.0%	24.9%	7.8

## Data Availability

The data presented in this study are openly available.
